# Quantitative analysis of tissue deformation dynamics reveals three characteristic growth modes and globally aligned anisotropic tissue deformation during chick limb development

**DOI:** 10.1242/dev.109728

**Published:** 2015-05-01

**Authors:** Yoshihiro Morishita, Atsushi Kuroiwa, Takayuki Suzuki

**Affiliations:** 1 Laboratory for Developmental Morphogeometry, RIKEN Quantitative Biology Center, Kobe 650-0047, Japan; 2 RIKEN Center for Developmental Biology, Kobe 650-0047, Japan; 3 Division of Biological Science, Graduate School of Science, Nagoya University Furo-cho, Chikusa-ku, Nagoya 464-8602, Japan

**Keywords:** Bayesian inference, Limb development, Quantitative analysis, Tensor analysis, Tissue deformation dynamics

## Abstract

Tissue-level characterization of deformation dynamics is crucial for understanding organ morphogenetic mechanisms, especially the interhierarchical links among molecular activities, cellular behaviors and tissue/organ morphogenetic processes. Limb development is a well-studied topic in vertebrate organogenesis. Nevertheless, there is still little understanding of tissue-level deformation relative to molecular and cellular dynamics. This is mainly because live recording of detailed cell behaviors in whole tissues is technically difficult. To overcome this limitation, by applying a recently developed Bayesian approach, we here constructed tissue deformation maps for chick limb development with high precision, based on snapshot lineage tracing using dye injection. The precision of the constructed maps was validated with a clear statistical criterion. From the geometrical analysis of the map, we identified three characteristic tissue growth modes in the limb and showed that they are consistent with local growth factor activity and cell cycle length. In particular, we report that SHH signaling activity changes dynamically with developmental stage and strongly correlates with the dynamic shift in the tissue growth mode. We also found anisotropic tissue deformation along the proximal-distal axis. Morphogenetic simulation and experimental studies suggested that this directional tissue elongation, and not local growth, has the greatest impact on limb shaping. This result was supported by the novel finding that anisotropic tissue elongation along the proximal-distal axis occurs independently of cell proliferation. Our study marks a pivotal point for multi-scale system understanding in vertebrate development.

## Abstract (precis)

**Summary:** Chick limb morphogenesis occurs through issue elongation independently of cell proliferation, with three tissue growth modes correlated with SHH expression.

## INTRODUCTION

A main goal in developmental biology is to understand how dynamic changes in organ morphology are realized as outcomes of coordinated molecular and cellular phenomena. To achieve this, clarifying the relationships among the dynamics at the molecular, cellular and tissue levels is required.

Vertebrate limb morphogenesis has been an attractive research subject for over 100 years. Over the last few decades, the genetic basis of limb morphogenesis has been well studied (Zeller et al., 2009). More recently, research trends have shifted from the elucidation of the genetic and molecular pathways controlling limb development to high-quality imaging of cellular behaviors. Different space- and direction-dependent cellular behaviors in developing limb buds have been reported; these include region-specific adhesion differences (Ide et al., 1998; Barna and Niswander, 2007), active cell migration (Li and Muneoka, 1999), oriented cell division (Gros et al., 2010; Sato et al., 2010) and asymmetric cell shape (Boehm et al., 2010), the roles of which in the unidirectional elongation of limb buds have been discussed. However, the extent to which individual cellular behaviors contribute to overall limb morphology is still unknown. This is owing to a large gap in spatial scale between such microscopic cellular behaviors (in the order of a micrometer) and the global morphology of the limb (on a millimeter scale), which consists of millions of cells. To fill the gap, what is critically needed is geometrical information about physical tissue deformation, that is, the macroscopic output of morphogenesis, which can be obtained only by constructing a quantitative tissue deformation map. The geometrical analysis of these maps would allow identifying when and where characteristic deformation patterns occur during the shaping of organs, which will provide a basis for the study of the inter-hierarchical relationships among molecular activities, cellular behaviors and tissue/organ morphologies.

In recent years, quantitative deformation analysis has become an active field of research, especially in studies using monolayer epithelial tissues of *Drosophila*, zebrafish and chicken embryos that are suitable for cellular-level time-lapse imaging by conventional confocal microscopy (Butler et al., 2009; Blanchard et al., 2009; Taniguchi et al., 2011; Voiculescu et al., 2007; Graner et al., 2008). In many vertebrate organs, however, *in toto* imaging or total cell recording by live imaging with high resolution is difficult, and only a few reports exist on the quantification of the dynamics of tissue deformation (Filas et al., 2008).

Regarding vertebrate limb development, there have been several reports on fate mapping using chick and mouse limbs with different tissue labeling methods (Vargesson et al., 1997; Clarke and Tickle, 1999; Sato et al., 2007; Pearse et al., 2007; Arques et al., 2007). Most of them have focused on comparison of the spatial distributions of labeled cells between stages separated temporally by several days, and have determined the origins of cells in anatomically different regions of a fully developed limb. In these studies, the distribution of markers observed at later stages was highly expanded and complex due to long-term tissue growth. Therefore, quantitative information on tissue deformation, i.e. the one-to-one relationship between positions of each unit of tissue before and after deformation, was difficult to extract precisely from the marker data. One of the few exceptions, in which the spatio-temporal pattern of tissue deformation characteristics was quantitatively analyzed, is the study by Marcon et al. (2011). The authors constructed a tissue deformation map for the mouse limb and estimated the spatio-temporal pattern of deformation characteristics based on the spatial distribution of clonal cells at different developmental stages and on some assumptions about tissue mechanical properties. The estimated map and deformation pattern were almost symmetric about the anterior-posterior (A-P) axis. By contrast, another lineage analysis by Harfe et al. (2004), using the *Shh::GFPCre/^+^;R26R/^+^* mouse, implied asymmetric expansion of tissues along the A-P axis, and descendants of SHH-expressing cells in the mouse limb bud give rise to a large proportion of the future autopod (specifically, digits 3/4/5), despite the restricted expression of *Shh* at the posterior end. The reason for this inconsistency is unknown, and, as the initial distribution of cells in the clonal analysis is unknown, the validity of the estimated deformation pattern cannot be addressed in statistical terms.

Recently, we developed a novel statistical method to construct tissue deformation maps of whole organs from limited space-time point data (Morishita and Suzuki, 2014). Applying this method, in this study we have constructed the first quantitative tissue deformation map for chick hindlimb development and extracted geometrical characteristics from the map that enable the study of inter-hierarchical relationships. It is easier to quantitatively measure the positions of injected landmarks before and after deformation in the chick than in the mouse. This allowed us to statistically evaluate the accuracy of the estimated spatio-temporal pattern of deformation dynamics, which is an advantage of our study over previous fate mapping studies and lineage analyses. Demonstrating that the spatio-temporal dynamics of deformation characteristics at the tissue scale are consistent with those of cell cycle and morphogen activity also validated the estimated deformation dynamics. In particular, we show that SHH signaling activity dynamically changes according to developmental stage and that this correlates strongly with the dynamic shift in tissue growth mode. Furthermore, by combining the resulting deformation map with morphogenetic computer simulations, we evaluated the roles of globally oriented local tissue stretching and spatially biased volume growth through cell proliferation in directional limb bud growth and limb-specific morphology.

Our study marks a pivotal point for multi-scale systems analysis in limb development. Moreover, our approach to the analysis of tissue deformation dynamics is not specific to the limb, and thus our study could serve as a model for vertebrate organ morphogenesis generally using practical lineage marking techniques.

## RESULTS

### Geometrical description of tissue-level deformation dynamics

**Fig. 1.**
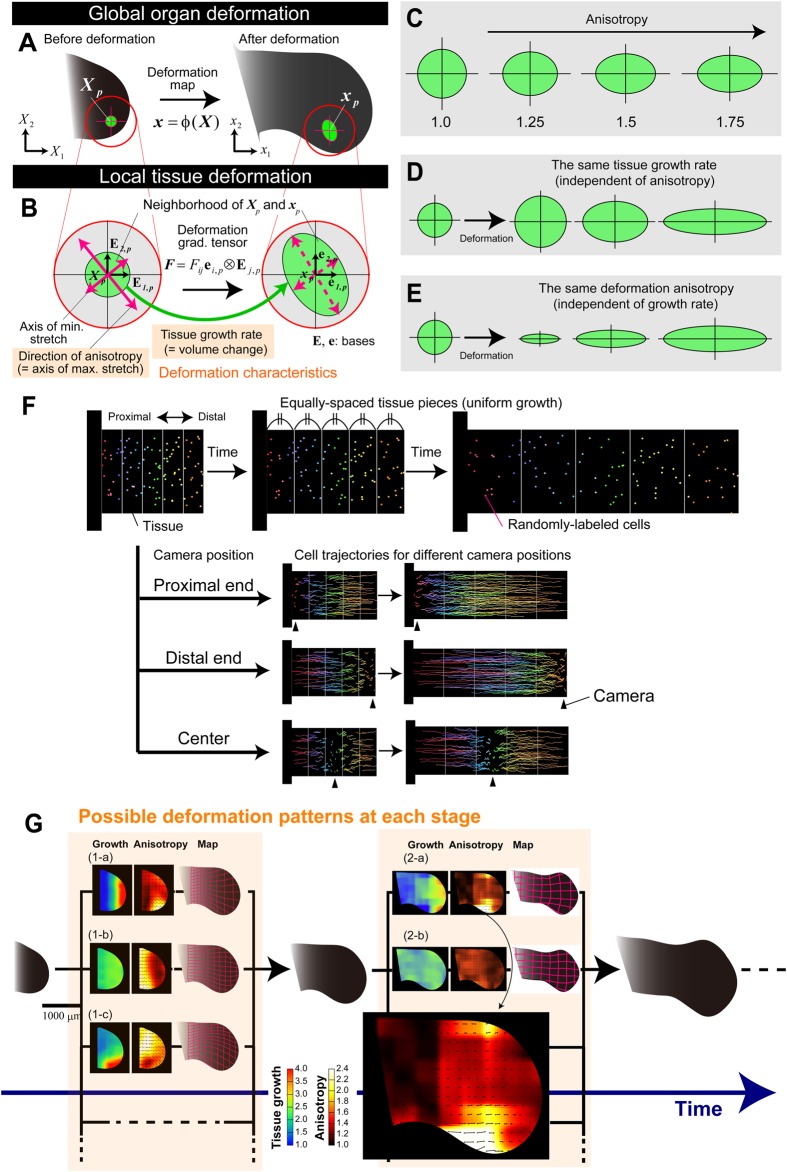
**Tissue-level characterization of deformation dynamics.** Global organ deformation is defined by map *ϕ* (A), and local tissue deformation is defined by the deformation gradient tensor ***F*** (B). Tissue growth rate and deformation anisotropy are two key characteristics. (C-E) Examples of local tissue deformation, with different values for deformation anisotropy, identical tissue growth rate and identical deformation anisotropy, respectively. (F) Cell trajectory or velocity in a growing tissue appears different, depending on the position of the camera, even if tissue deformation dynamics are identical. Here, the case of spatially uniform growth is shown. (G) Possible scenarios for deformation dynamics at each stage (artificially generated by computer simulations). For each stage, different deformation dynamics, producing the same morphology, are possible. The heat maps indicate spatial patterns of tissue growth rate (left) and deformation anisotropy (right). Black arrows represent the orientation of the principal stretch at each position [see the higher magnification view in case (2-b) for an example]. In case (1-a), the morphology in the middle panel is achieved by distally biased volume growth and anisotropy of tissue deformation along the proximo-distal axis. In case (1-b), spatially uniform volume growth and proximally biased anisotropic tissue deformation generate the same morphology, and in case (1-c), the same morphology is also realized by a more complex deformation pattern with posteriorly biased tissue growth. The deformation dynamics can change with developmental stage [cases (2-a) and (2-b)].

In characterizing the macroscopic deformation patterns of tissues that are composed of a large number of cells, it is useful to regard them as continuums, by neglecting or averaging out the variability in the shapes and sizes of individual cells. In the case of the chick limb bud, there are millions of cells, and thus the continuum approach is appropriate. When an organ is regarded as a continuum, its deformation is mathematically described as a map (Bonet and Wood, 2008), i.e. the relationship between the spatial coordinates of each unit of tissue or landmark (e.g. small populations of cells) before and after deformation (Fig. 1A; Materials and Methods). Such a positional relationship, of course, might to some extent be different between embryos. The map we refer to here is an average over multiple embryos; smaller variability in tissue deformation between embryos from the average map indicates that the morphogenetic process is more robust and deterministic.

Once the map is quantified, local tissue deformation can be characterized by using a deformation gradient tensor calculated at each location (Fig. 1B). Intuitively, the tensor describes how a small circle [in a two-dimensional (2D) scenario] or sphere [in a three-dimensional (3D) scenario] surrounding the focal position deforms during a given time interval. Two quantities calculated from the tensor components define the local deformation. One is the ‘tissue growth rate’, which is a scalar quantity and defines changes in area or volume around the focal position (Fig. 1B). We stress that the tissue growth rate is a quantity at the tissue scale; whereas, at the cellular level, the cell proliferation rate is the major determinant of the tissue growth rate. We note that other factors, such as change in cell size and secretion of extracellular matrix, might also be involved. Thus, clarifying which cellular process contributes most to the tissue growth rate at each position in the tissue and at each developmental stage is important for linking events with different spatial scales, i.e. tissue-level deformation and cellular-level activities.

The other deformation characteristic is a vector quantity referred to as ‘deformation anisotropy’ – in other words, directionally biased local deformation of tissue (Fig. 1B). For example, in a 2D scenario, anisotropy is expressed by the vector along which maximal elongation occurs, and the magnitude of which is the ratio of maximum to minimum elongation (along the perpendicular axis) (Fig. 1B). The magnitude of anisotropy for isotropic deformation is set to 1, and its value becomes larger for deformation with a larger bias (Fig. 1C). Note that the tissue growth rate and the deformation anisotropy are independent of each other; even if one remains constant, overall deformation is different if the other varies (Fig. 1D,E). At the cellular level, deformation anisotropy can be interpreted as cell rearrangement that is caused by the total effect of space- and direction-dependent cellular behaviors, such as oriented cell division and region-specific adhesion differences.

### Cell velocity fields calculated from cell tracking data cannot be immediately linked to tissue deformation characteristics

In recent reports, the velocity fields within growing limb or fin buds in early development were quantified from short-period cell-tracking data, and, based on these velocity fields, cell motility and directional elongation were discussed (Gros et al., 2010; Wyngaarden et al., 2010; Hopyan et al., 2011). To avoid confusion between the results in the previous studies and those in this study (shown below), we would like to clarify the following two points: first, cell velocities calculated from cell-tracking data cannot be directly linked to tissue deformation dynamics and cell motility. Second, the vector of velocity measured in previous studies and that of deformation anisotropy quantified in this study are completely different quantities. These can be understood by considering the following, simple thought experiment. Consider time-lapse imaging of cell trajectories in the case of spatially uniform one-dimensional growth, and thus, each part of the tissue growing at the same rate (please note that the following discussion also holds for cases with arbitrary non-uniform growth) (see Fig. 1F; supplementary material Movies 1-6 and Appendix S1 for a more rigorous explanation with equations). Then consider how the trajectories or velocities of randomly distributed landmarks (or cells) appear with the growth of tissue. As shown in Fig. 1F, they look different, depending on the position of the camera (i.e. the coordinate system adopted): if the camera is fixed at the leftmost or proximal end, the velocity becomes higher for cells located farther to the right or more distally (Fig. 1F, top), and vice versa (Fig. 1F, middle). If the camera is fixed so as to focus on the cell located at the center of the tissue, the velocity becomes maximal at the two ends of the tissue (Fig. 1F, bottom). As each of these observations are for the same tissue deformation dynamics, i.e. uniform growth, the example given above clearly shows that a difference in the spatial pattern of cell velocity measured in a growing tissue does not mean a difference in tissue deformation dynamics. Without going into detail, to connect the velocity field to tissue deformation dynamics, the spatial derivative of velocity field (specifically, velocity gradient tensor) needs to be calculated (Bonet and Wood, 2008).

For the same reason, cell motility cannot be discussed only from the cell velocity field measured in a growing tissue. For instance, in Fig. 1F (top), is it correct to say that the motility of cells located at the distal side is higher? It is not, of course, although in a non-growing tissue, cell velocity directly reflects cell motility.

We should also note that, in this study, active cell migration is neglected for the following reasons: observations of sections of mesenchymal tissue showed tight packing of mesenchymal cells, and the tracking data for fluorescent markers showed invariance of neighbor relationships among the markers (during at least 12 h).

### Analysis of tissue deformation dynamics can identify the positions and times at which distinctive deformations occur

As shown in the literature (e.g. see Prusinkiewicz and Runions, 2012; Marcon et al., 2011; Morishita and Suzuki, 2014), there are infinite ways of mapping to generate the same organ morphology (i.e. the outline of an organ) from the same initial shape. In principle, limb-specific morphology and unidirectional outgrowth of the limb bud can be realized by multiple, different deformation dynamics (Fig. 1G). Furthermore, such deformation patterns can change drastically as the limb develops (Fig. 1G). Clear shifts in deformation patterns imply the existence of multiple deformation modes and multiple molecular/cellular factors that regulate deformation characteristics at each mode and at the transitions between modes.

In order to find out which of the many possible deformation dynamics occurs in living tissues, it is vital to accurately capture the spatial heterogeneity of deformation characteristics, especially regions with higher growth rates and/or anisotropy of tissue stretching, which we call ‘morphogenetic hot spots’ (Morishita and Suzuki, 2014). Such morphogenetic hot spots must exist in order to generate organ-specific shapes, as otherwise only isotropically expanded, ball-like structures would form. Once these hot spots are identified, inter-hierarchical relationships between events at different spatial scales will be revealed by analyzing the correlation between tissue-level deformation and the molecular/cellular activities occurring there (e.g. morphogen expression and intercellular positional rearrangement).

### Constructing deformation maps for chick hindlimb development from spatially and temporally limited landmark data

To obtain data for the creation of deformation maps, one approach is to use total cell recording by live imaging. However, this is often difficult because of large tissue size and opaqueness. Therefore, we employed a different approach that depends on discretized snapshot data, which we obtained from simple fluorescent dye marking.

**Fig. 2.**
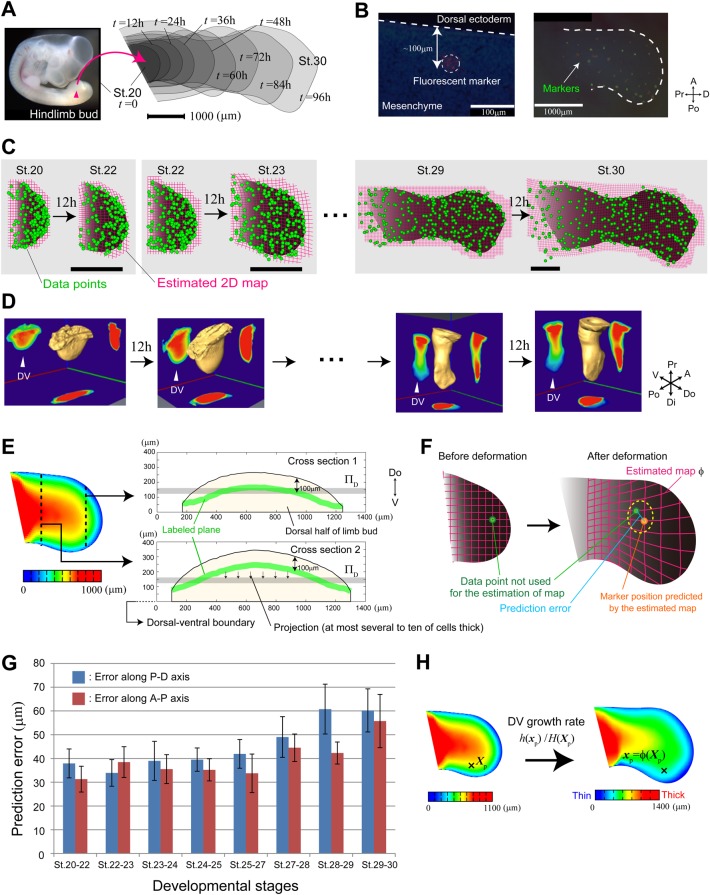
**Estimation of tissue deformation dynamics for chick hindlimb development from dye-marking data.** (A) Dynamic changes in chick hindlimb morphology from stage 20 to stage 30. (B) Between 20 and 50 markers were randomly injected into the mesenchyme (right) at a depth of ∼100 µm from the dorsal ectoderm (left). (C) From positional data on markers in the AP-PD plane (green circles), the 2D deformation map of the dorsal half of a limb bud was estimated for each 12-h time interval (magenta lattice). The number of markers for estimating the map for each time interval was: *n*=140 (stage 20-22), *n*=203 (stage 22-23), *n*=224 (stage 23-24), *n*=288 (stage 24-25), *n*=231 (stage 25-27), *n*=290 (stage 27-28), *n*=306 (stage 28-29), *n*=321 (stage 29-30). Scale bars: 1000 µm. (D) Spatial pattern of D-V thickness was measured with an OPT scanner for each developmental stage (every 12 h). (E) Although not all data were necessarily on the frontal plane (Π_D_) on which the 2D-deformation map is estimated, the distance between the labeled plane (green) and Π_D_ (gray) is short, enabling us to estimate precisely the map by using the data from markers after projecting them to Π_D_. (F) The goodness of estimation can be evaluated as the prediction error for the estimated maps (cross-validation). (G) Prediction errors of estimated maps along the P-D and A-P axes for different developmental stages. (H) The growth rate along the D-V axis at each point ***X***_p_ can be approximately calculated as *h*(*ϕ*^2D^(***X***_p_))/*H*(***X***_p_).

**Fig. 3.**
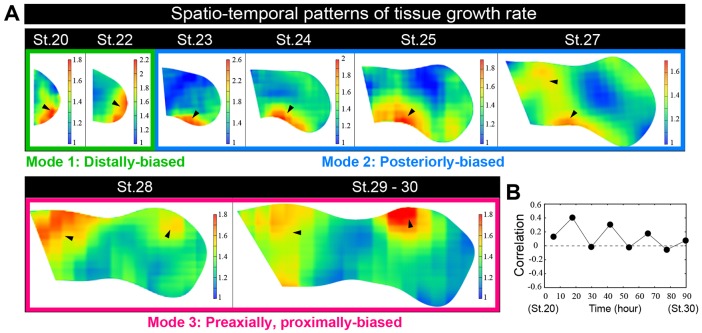
**Quantitative geometrical analysis of chick hindlimb morphogenesis reveals three characteristic tissue growth modes.** (A) Spatio-temporal patterns of tissue growth rate per 12-h interval. Black arrowheads indicate regions of high tissue growth rate. (B) Correlations between area and D-V growth rates.

We recently developed a novel method that combines snapshot lineage tracing with Bayesian statistical estimation to construct whole-organ deformation maps from limited space-time point data (Morishita and Suzuki, 2014). In this method, a regular lattice enveloping the target organ before deformation is considered, and how the lattice deforms over a given time interval is estimated. Except for positional data for landmarks before and after deformation, all that is required is the biologically plausible assumption that deformation of tissue occurs smoothly. As shown below, applying the method to data for chick limb development, we here estimated the deformation map with high precision and analyzed the deformation dynamics (Figs 2, 3 and 7).

### 2D deformation map for the frontal plane of the dorsal region of a limb bud

Clonal analysis using a mouse limb bud showed that the dorsal region of the limb bud does not intermingle with the ventral region (Arques et al., 2007). Thus, we first examined the 2D deformation dynamics of the dorsal half on the frontal plane (defined by the proximo-distal and anterior-posterior axes) at an intermediate depth in the dorsal half during chick hindlimb development (Fig. 2A). For tissue labeling, we used the fluorescent dye combination DiI/DiO (Vargesson et al., 1997). Due to limitations in deep imaging and precise depth control in labeling, the markers (with a diameter of approximately several tens of micrometers) were randomly injected into the mesenchyme at a depth of ∼100 µm from the dorsal ectoderm (Fig. 2B; Materials and Methods; supplementary material Appendix S2), and the position of each marker was measured by electric fluorescence stereoscopic microscopy before and after (i.e. 12 h after) deformation (Fig. 2B). To estimate the map for each interval, data from 7 or 8 embryos (representing a few hundred markers in total) were used (Fig. 2C). As shown later, even if the number of points marked in each embryo is limited (e.g. a few tens of points), using snapshot data from multiple independent embryos enables reliable and reproducible estimation of the deformation map and deformation characteristics. Measurements of marker positions were performed over eight consecutive time intervals (from stage 20 to stage 30).

To evaluate the relative positions of injected markers (at a depth of 100 µm) along the dorsal-ventral (D-V) axis, using optical projection tomography (OPT) (Sharpe et al., 2002), we measured the spatial pattern of D-V thickness of the limb bud every 12 h (Materials and Methods; Fig. 2D and supplementary material Fig. S1). Although the D-V thickness depends on the position within the limb bud and although it changes with time, the thickness of the dorsal half ranges from 100 µm to 350 µm at almost all positions before stage 27. Therefore, taking plane Π_D_ at a depth of ∼150-200 µm (Fig. 2E), we can estimate precisely enough the average 2D deformation map on the frontal plane of the dorsal half of the limb bud by using the data from markers after projecting them to Π_D_. Although not all data were necessarily on the target plane Π_D_, the distance between the labeled plane (shown by the green lines in Fig. 2E) and Π_D_ is short (specifically, the size of several to ten cells on average); it is highly unlikely that the 2D deformation pattern drastically changes along the D-V axis within the thickness of several cells. Furthermore, as shown later, the correlation between the growth rate along the D-V axis and the 2D area growth rate on the frontal plane is low, regardless of developmental stage, ensuring that the geometrical analyses of tissue deformation dynamics carried out in this study precisely capture the average deformation pattern of the dorsal half of the limb bud.

At later stages (i.e. after stage 27), there is a significant influx of muscle cells (Ros et al., 2003; Christ and Brand-Saberi, 2002), and the D-V thickness increases, especially in the zeugopod region, meaning that the estimated 2D deformation map in plane Π_D_ might be slightly different from the average deformation pattern of the dorsal half. By contrast, in the autopod region, the D-V thickness of the dorsal half is still small (200-400 µm), and thus the estimated map is considered to precisely reflect the true average pattern.

The 12-h interval used for the analysis of tissue deformation dynamics is short enough to allow precise capture of the position of each marker, even though the markers expand slightly with tissue growth. An important point is that, in order to quantify local deformation at a given focal point, we do not need information about the change in marker size itself. Rather, we need to know how the relative positions of neighboring markers change over time.

We then evaluated the accuracy of the estimated maps by their predictive performances (cross-validation) (Fig. 2F; supplementary material Appendix S3). The size of prediction errors was ∼40-50 µm on average (Fig. 2G), which is much smaller than the scale of the mesh size (150-250 µm) used for the estimation of maps, thus demonstrating the accuracy of the estimated maps in capturing quantitatively the dynamic change in limb morphology. The residual errors in the estimate were also calculated and confirmed to be small enough (∼50 µm) at each developmental stage. As the estimation process for each time interval included data from several embryos, the small error sizes also indicated that the morphogenetic process was highly consistent among embryos.

### Evaluation of growth rate along the dorsal-ventral axis

We quantified the 2D deformation map ***x***=*ϕ*^2D^(***X***) on frontal plane Π_D_. As stated before, accurately measuring the positional change of each fluorescent marker along the dorso-ventral (D-V) axis in live organs is difficult, and thus it is also difficult to examine how the 2D deformation map on the frontal plane depends on D-V depth. Assuming that the dependence of the 2D deformation map on depth is not large, due to the flatness of the limb bud, we here approximately evaluated the growth rate along the D-V axis as *h*(*ϕ*^2D^(***X***_p_))/*H*(***X***_p_) at each point ***X****_p_*, where *H(**X**)* and *h(**x**)* are spatial patterns of D-V thickness before and after deformation measured with OPT (Fig. 2D). Statistical analysis showed that the correlation between 2D deformation on the frontal plane and D-V growth rate is low (around 0.2), regardless of stage (Fig. 3B), indicating that these two deformation processes can be dealt with independently and that the estimated 2D deformation maps precisely capture the average deformation dynamics on the frontal plane of the dorsal half of the limb bud, at least during stage 20-30, (supplementary material Appendix S4). We also evaluated 3D volume growth rate by multiplying the area and D-V growth rates at each position (Fig. 2H) and found that the spatial patterns of 2D area growth and 3D volume growth are very similar (supplementary material Fig. S2).

### Geometrical analysis reveals three characteristic tissue growth modes

Our mapping analysis of chick limb deformation revealed that the region with the highest growth rate changed over time. During stages 20-30, three distinct growth modes were identified. In Fig. 3A and supplementary material Fig. S2, the spatio-temporal patterns of area and volume growth rate over 12 h are shown as heat maps, with warmer colors indicating higher growth rates. Tissue growth was (i) distally biased during early stages (stages 20-23) (see black arrowheads in Fig. 3A), (ii) posteriorly biased during middle stages (stages 23-28), and (iii) pre-axially and proximally biased during later stages (stages 28-30).

### Tissue-level volume growth pattern is consistent with spatial heterogeneity of cell cycle

**Fig. 4.**
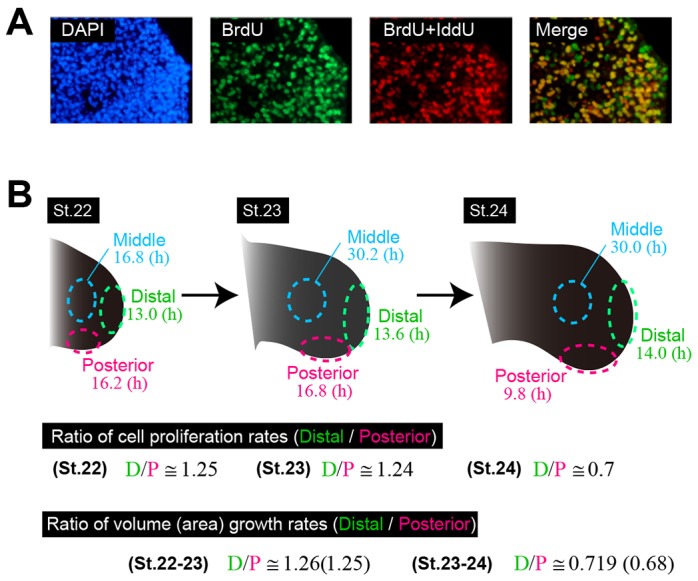
**Spatial patterns of tissue growth rate can be explained by the spatial heterogeneity of cell cycle time.** (A) BrdU/IddU incorporation at the distal side at stage 23. (B) Cell-cycle time was measured at three locations at stages 22, 23 and 24. The ratio of cell proliferation rates (1/cell cycle time) between distal and posterior regions is quantitatively consistent with that of tissue-level volume (or area) growth rates. For calculating the cell cycle time at each location and at each stage, 600 cells from three embryos were used.

We next examined whether these three modes of tissue growth are quantitatively consistent with the spatio-temporal dynamics of cell cycle and morphogen activity. By pulse-chase labeling with BrdU/IddU (Martynoga et al., 2005) (Fig. 4A), we measured cell cycle times in three regions (specifically, posterior, distal and middle regions) at three consecutive stages (stages 22, 23 and 24) (Materials and Methods; supplementary material Appendix S5). The ratio of cell proliferation rate in the distal and posterior regions was found to be quantitatively consistent with that of the tissue growth rate (i.e. both volume and area expansion rate) (Fig. 4B). Specifically, both ratios were ∼1.25 at stage 22/23, and then changed to ∼0.7 at stage 24. This means that the growth rate at the tissue scale can be explained mainly by the proliferation rate at the cellular scale.

### Transition of tissue growth mode is strongly correlated with time-variant SHH signaling activity

**Fig. 5.**
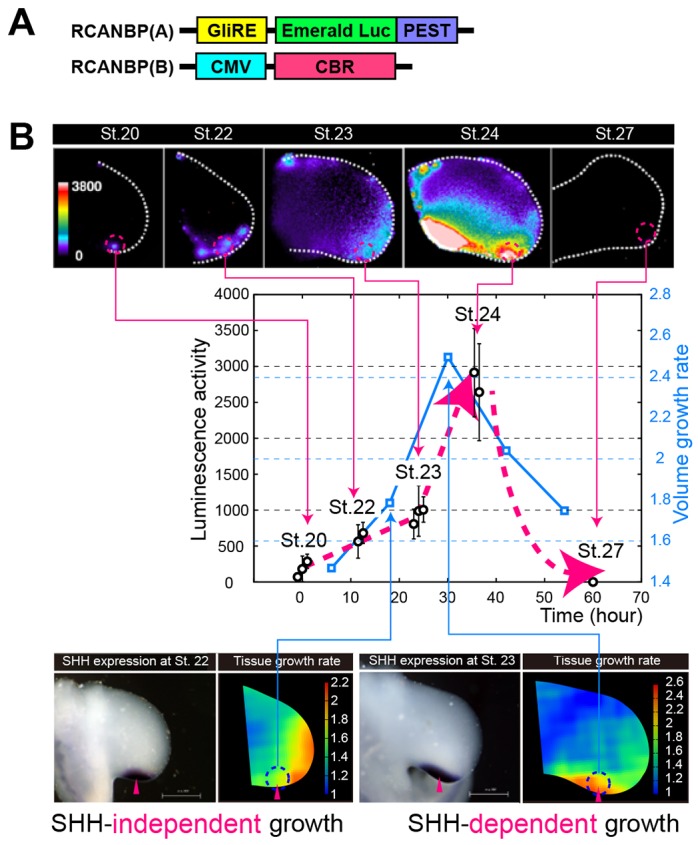
**Dynamic change in SHH signaling activity during limb development.** (A) We detected SHH signaling activity using emerald luciferase downstream of the Gli promoter and normalized the activity relative to red luciferase activity, driven by the CMV promoter in the same limb bud. (B) Normalized luminescence activity at a posterior region for different developmental stages (open circles). Whereas signaling is at a low level only in the posterior side at stage 20, after stage 23 it is sharply increased with expansion of the activated area, which is consistent with the timing of growth mode shift from distally biased to posterior biased. SHH signaling disappears by stage 27. The blue line indicates the dynamics of volume growth rate in the posterior region.

**Fig. 6.**
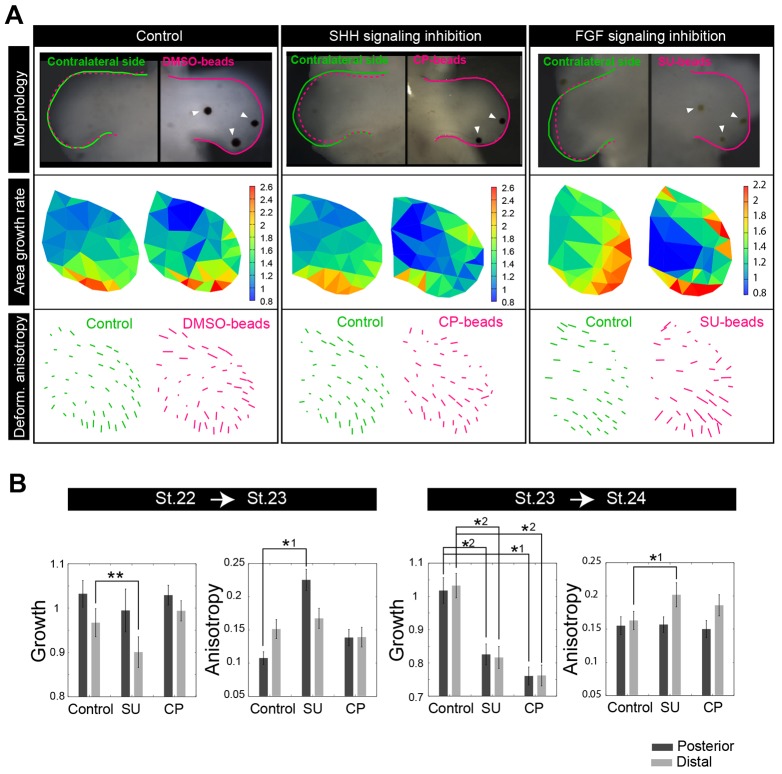
**Contributions of SHH and FGF signaling to deformation characteristics.** (A) Typical examples of effects of bead implantation; from top to bottom, morphology at 12 h after implanting beads, spatial patterns of area growth rate and deformation anisotropy are shown. The area growth rate and deformation anisotropy are calculated for both beads and control. From left to right, the results for implanting beads soaked with DMSO (control), cyclopamine (CP) and SU5402 (SU) are shown. On the contralateral side (left limb bud), no beads were implanted. (B) Summary of the effects of inhibiting SHH and FGF signaling. For two different time intervals (stage 22-23 and stage 23-24) and different regions (posterior and distal), the change in tissue growth rate and the deformation anisotropy are calculated. Error bars indicate s.e. *1: *P*<0.05 (Welch's *t*-test); *2: *P*<0.05 (Student's *t*-test); **: *P*<0.1 (Student's *t*-test).

Previous studies have suggested that several types of growth factor signals play key roles in the control of growth and patterning of the limb (Zeller et al., 2009; Mariani et al., 2008; Riddle et al., 1993; Niswander et al., 1994; Laufer et al., 1994; Niswander and Martin, 1993; Prykhozhij and Neumann, 2008; Xu et al., 2000). Among them, SHH is implicated in generating differences along the A-P axis and was here examined experimentally. We first established an efficient and sensitive method to quantify SHH signaling activity directly in the limb bud (Fig. 5A). We infected limb buds with viruses to express emerald luciferase under the control of the RCANBP(A)Gli-responsive element and, for normalization of expression, red luciferase under the control of the RCANBP(B)CMV promoter. Using a luminescence microscope, we directly quantified photons from emerald luciferase and red luciferase luminescence independently with different band path filters in the infected limb bud (Materials and Methods; supplementary material Appendix S6). Importantly, we found that SHH signaling activity dramatically changed over space and time, although the expression pattern of *Shh* by *in situ* hybridization looked the same, independent of stage (from stage 18 to stage 25). Until stage 22, weak SHH signaling activity was seen only in a restricted area of the posterior edge, near the *Shh* expression region (Fig. 5B). Around stage 23, SHH signaling activity rapidly increased on the posterior side, expanded into more anterior regions, and then completely disappeared by stage 27. In particular, as shown in Fig. 5B, the dynamics of SHH signaling activity in the posterior region was strongly correlated with that of tissue growth rate there, indicating a clear connection between the dynamic shift of tissue growth mode and the dynamics of SHH signaling activity. Such a connection is supported by an experiment in which SHH activity was inhibited through cyclopamine-soaked bead implantation (Fig. 6A; supplementary material Fig. S3; Materials and Methods). Although inhibition of SHH signaling by cyclopamine (CP) had no effect on posterior and distal growth during the interval from stage 22 to stage 23 (distally biased mode), it caused a clear decrease in the tissue growth rate during the interval from stage 23 to stage 24 (posteriorly biased mode) compared with the control (Fig. 6B), when SHH signaling activity increases and expands (Fig. 5B). This also indicates that, although *Shh* expression is observed at the posterior end from earlier stages (specifically, from stage 17), its expression level and/or downstream signaling activity dynamically changes over time, causing a temporal dependence of the effects of SHH signaling on the tissue growth rate.

The asymmetric growth pattern along the A-P axis through SHH-dependent posterior expansion is consistent with the fact that descendants of SHH-expressing cells in the mouse limb bud give rise to a large proportion of the future autopod (Harfe et al., 2004).

In contrast to the SHH case, inhibition of FGF signaling by SU5402 (SU) caused a decrease in the tissue growth rate independently of developmental stage; therefore, the distally biased growth mode observed in earlier stages is determined by FGF signaling from the apical ectodermal ridge. On the other hand, SHH-independent digit 1 growth and muscle cell migration and/or proliferation are the most likely contributors to the third mode of tissue growth, from stage 28 to stage 30 (Ros et al., 2003; Christ and Brand-Saberi, 2002).

### Local deformation anisotropy is globally aligned along the P-D axis

**Fig. 7.**
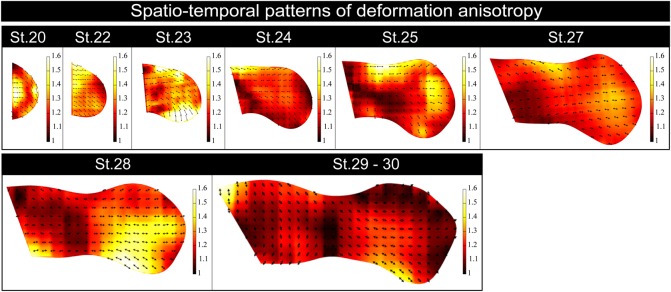
**Local deformation anisotropy is globally aligned along the P-D axis.** Spatio-temporal patterns of deformation anisotropy per 12-h interval are expressed as heat maps. The black double-headed arrows indicate the directions of deformation anisotropy vectors; their lengths are proportional to the magnitudes of the anisotropy, which is also shown in different colors.

Besides differential dynamics in local growth, our mapping analysis revealed an intriguing pattern of ‘deformation anisotropy’ (i.e. local bias in tissue elongation); as shown by the double-headed arrows in Fig. 7, we found that local anisotropies are globally aligned along the proximo-distal (P-D) axis independently of developmental stage, and that tissue elongation rate has no large bias along the P-D axis. These findings support the hypothesis proposed by Boehm et al. (2010), based on mechanical simulations, that unidirectional elongation of the limb bud cannot be achieved with spatially biased tissue growth alone. Furthermore, these findings point to the possibility that the global orientation of local deformation anisotropy might be the most important factor in determining overall morphogenesis.

### Globally aligned local deformation anisotropy, not local growth, has the greatest impact on limb shaping

**Fig. 8.**
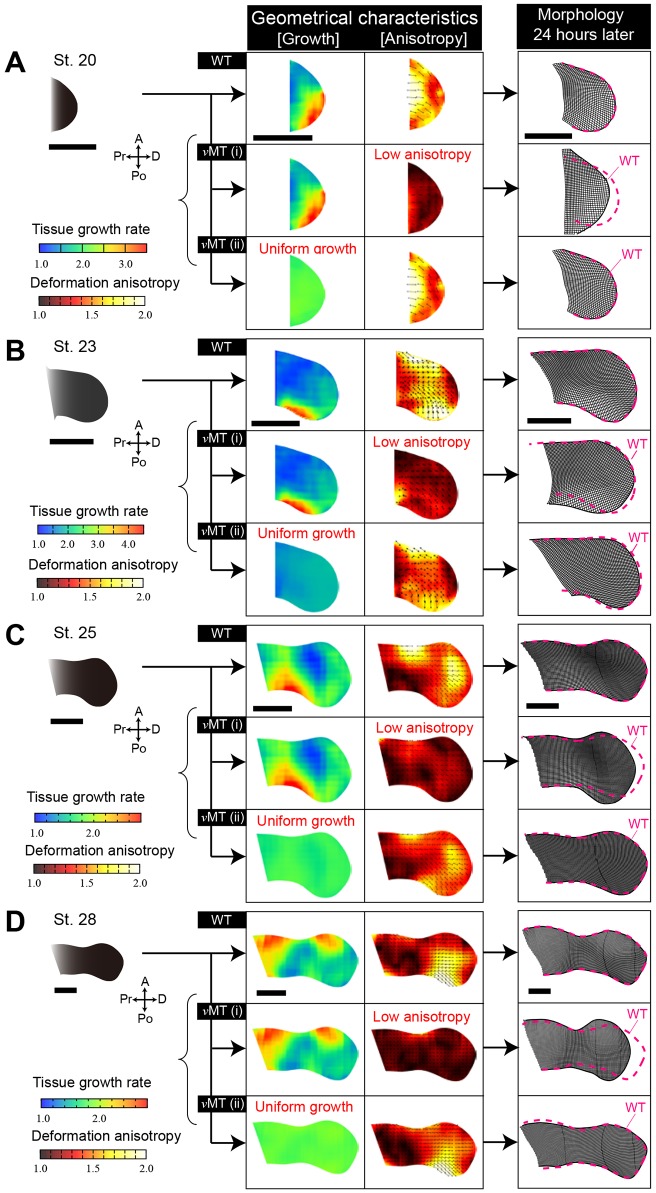
**Morphogenetic simulation reveals that globally oriented local tissue stretching, not spatially biased volumetric growth, is the major determinant of directional limb bud elongation and limb-specific shaping.** In each panel (A-D), the top subpanels show the morphological change in a wild-type (WT) limb bud over 24 h. The middle subpanels show the morphological change in the limb bud of virtual mutant I [*v*MT(i)], in which the growth pattern is the same as in WT, but where the deformation anisotropy is low across the whole limb bud. The bottom subpanels show the limb bud of virtual mutant II [*v*MT(ii)], which has a pattern of anisotropy similar to that of WT but no spatially biased growth. Scale bars: 1000 µm.

To test this possibility, we performed morphogenetic simulations for two limit situations (Fig. 8; supplementary material Fig. S4 and Appendix S7): (I) the situation in which the spatial pattern of tissue growth rate is the same as that in normal development but anisotropy is low over the whole limb bud; and (II) the situation in which the spatial pattern of anisotropy is similar to that in normal development but the tissue growth rate is uniform. For simulations, we developed a modified version of the vertex dynamics model that is widely used in multicellular simulations (Honda et al., 2004; Farhadifar et al., 2007). A major difference between our modified vertex model and most other vertex models is that we do not include assumptions about tissue mechanical properties; instead, the energy function that determines the dynamics of vertices includes only geometrical constraints so as to realize the situations I and II described above.

The results were clear: for all developmental stages, the directional growth and characteristic shaping (such as formation of paddle-like autopod shapes), with proper size observed in normal chicken hindlimb development, could not be reproduced using only similarities in tissue growth pattern. By contrast, a pattern similar to wild-type deformation anisotropy was sufficient to explain these morphogenetic processes without spatially biased growth. Note that in the latter simulation, the tissue growth rate at each position was set to be the average value over the whole limb bud of the wild type, in which morphogen-dependent growth is reflected. When the rate was changed, the whole size changed, but the shape did not, indicating that morphogen-dependent growth is important in the regulation of organ size.

### Anisotropic tissue elongation along the P-D axis occurs independently of cell proliferation

**Fig. 9.**
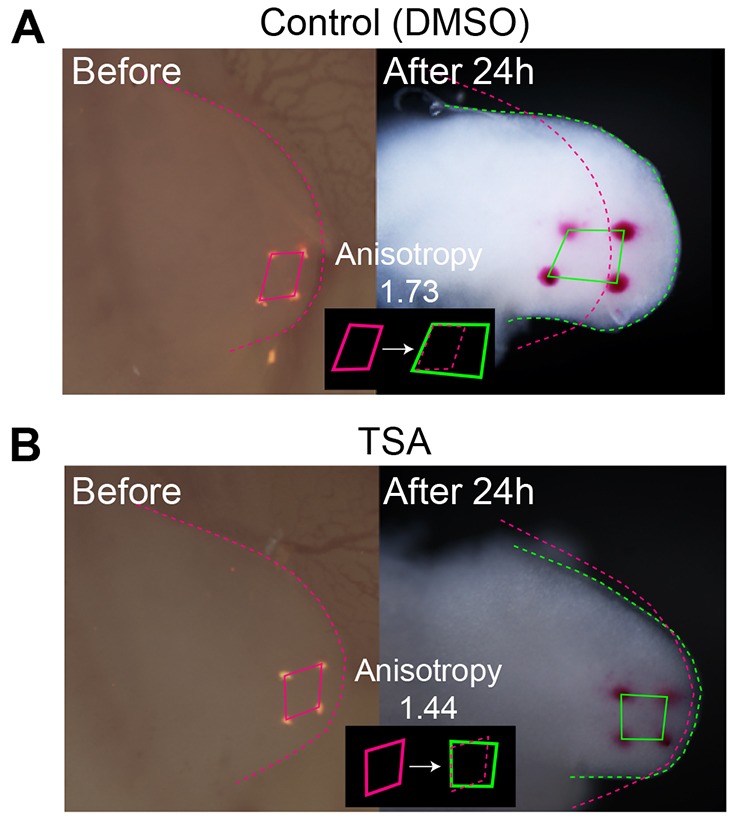
**Anisotropic tissue deformation was observed even in the absence of cell proliferation.** (A) Control, and (B) trichostatin (TSA) treatment.

Although the cause of anisotropic tissue elongation along the P-D axis is still unknown, we found that it occurs even in the absence of cell proliferation (Fig. 9; supplementary material Fig. S5; Materials and Methods). In the chick limb, cells entering S phase can be inhibited using trichostatin A (Towers et al., 2008) and monitored by BrdU incorporation (supplementary material Fig. S5). We transplanted several small crystals of DiI to the mesenchyme of the distal half of limb buds and calculated the deformation anisotropy from the change in relative positions of the transplanted DiI crystals over 24 h; average anisotropy was 1.55 among the three embryos treated with trichostatin A, whereas it was 1.74 among the three embryos treated with DMSO (control). This experiment demonstrated that deformation anisotropy can be generated independently of cell proliferation.

### FGF signaling as a regulator of deformation anisotropy

We also examined how the inhibition of SHH signaling or FGF signaling affects deformation anisotropy at distal and posterior regions. We observed that the inhibition of FGF signaling by SU5402 perturbed the deformation anisotropy, whereas SHH signaling inhibition by cyclopamine scarcely affected it (Fig. 6B).

## DISCUSSION

In order to understand how molecular- and cellular-level phenomena are reflected in global organ morphology, the construction of quantitative tissue fate maps – macroscopic, tissue-level information – is crucial as a first step. In this study, applying a recently developed method for estimating tissue deformation dynamics from spatially and temporally limited dye-marking data to chick limb development, we generated a precise tissue deformation map and determined the spatio-temporal patterns of deformation characteristics; the accuracy of estimated quantities was statistically guaranteed through cross-validation.

As shown in the experiments in which morphogen signaling and cell proliferation are inhibited by bead implantation during chick hindlimb development, the estimated deformation map for normal hindlimb development has enabled us to determine when, where and to what extent specific molecular and cellular phenomena correlate with deformation characteristics. Our analysis shows quantitatively the dynamic relationship between morphogen activity and tissue-level deformation characteristics in vertebrates. Specifically, the quantitative consistency between the spatio-temporal dynamics of tissue growth (i.e. the shift in growth mode) and SHH signaling activity is intriguing and should merit intensive future investigation. This finding demonstrates the importance of analyzing the dynamic nature of morphogen signaling activities, and not merely static ON/OFF states of their expression patterns, for understanding the inter-hierarchical relationships between molecular activity and tissue-level morphogenetic change.

Importantly, SHH is an essential factor for digit patterning, and a large proportion of the cells in the future autopod region are derived from the posterior, SHH-affected region in the early limb bud (Riddle et al., 1993; López-Martínez et al., 1995; Chiang et al., 2001). This raises the question of how the dynamic nature of SHH signaling in the posterior region is connected to the evolutionary origin of digits and/or Turing-type digit formation (Sheth et al., 2012; Raspopovic et al., 2014).

Quantitative estimation of tissue deformation dynamics allows evaluation of the contributions of distinct driving mechanisms that coexist during organogenesis. In this study, by combining estimated deformation maps and morphogenetic simulations (geometrical simulations without any assumptions about tissue mechanical properties), we showed that globally oriented local tissue stretching, not spatially biased volume growth, is the main determinant of directional limb bud growth and limb-specific shaping. We also found that there was not a large bias in the rate of elongation along the P-D axis, meaning that the limb field uniformly expands during its development along this axis. Interestingly, the experiment using trichostatin A showed that the anisotropic tissue elongation we observed is independent of cell proliferation, thus strongly suggesting a mechanism other than spatial heterogeneity of tissue growth or cell cycle length. As mentioned before, based on mechanical simulations, Boehm et al. (2010) suggested that mechanisms other than spatially biased tissue growth are necessary for achieving unidirectional elongation of the limb bud. Our results from quantitative analysis of tissue deformation dynamics with a clear statistical criterion and newly developed geometrical simulations have confirmed this suggestion.

As for a molecular mechanism, bead transplantation experiments showed that the inhibition of FGF signaling perturbed the deformation anisotropy, which suggests the involvement of FGF signaling for unidirectional limb bud elongation. On the other hand, as Gros et al. (2010) reported that non-canonical Wnt signaling is related to the orientation of cell division, we performed experiments in which JNK was inhibited by implanting beads soaked with SP600125 or by directly adding the inhibitor over the hindlimb *in ovo* (Gros et al., 2010; Nissim et al., 2007). In contrast to the experiments in which FGF was inhibited, there were no noticeable changes in whole-limb morphology during the 24 h following implantation in most (9 out of 10) of the embryos (data not shown).

As described before, recent cell biology studies have revealed space- and direction-dependent cellular behaviors, but it is still unknown which cellular behaviors have the most significant effect on tissue-level deformation anisotropy. To address this issue, it will be important to quantitatively determine how each behavior is correlated with the direction and magnitude of anisotropy at each position and at each developmental stage identified in this study. As a possible mechanism for anisotropic tissue deformation, some kinds of mechanical constraint along a specific axis might be involved as well. It is notable that elongation of the *Drosophila* wing blade along the P-D axis is driven by a contractile force in the hinge region (Aigouy et al., 2010). Anisotropy in stress distribution in either mesenchyme or epithelium in the vertebrate limb bud could induce such a unidirectional elongation. It would be interesting to examine in future work how the formation of cartilage and its directional growth are related to anisotropic tissue deformation (Chimal-Monroy et al., 2003; Gao et al., 2011).

Our approach of geometrical analysis of tissue-level deformation dynamics will contribute to multi-scale understanding of organ morphogenesis. Furthermore, extracting and comparing distinctive deformation characteristics, i.e. morphogenetic hot spots, in homologs among different species should provide quantitative guideposts for discussing morphological diversity and evolution between species.

## MATERIALS AND METHODS

### Deformation map and deformation gradient tensor

The global deformation of organ morphology is described by a map ***x***=*ϕ*(***X***), where ***X*** and ***x*** are positions of each piece of tissue before and after deformation, respectively. The deformation gradient tensor, ***F*** (*F_ij_*=∂*φ_i_*/∂***X****_j_*), includes all information about local tissue deformation around each position. ***F*** is decomposed into ***F=RU*** (***R***, rotation tensor; ***U***, right stretch tensor). Tissue growth rate is defined as det[***F***]. Deformation anisotropy is a vector, and its magnitude and direction are defined as *λ*_1_/*λ*_2_ and ***v***_1_/||***v***_1_||respectively, in which *λ*_1_ and *λ*_2_ (*λ*_1_≥*λ*_2_) are eigenvalues of ***U***, and ***v***_1_ is the eigenvector for λ_1_.

### Data for quantifying tissue deformation dynamics

For estimating 2D deformation map, we measured the positional changes of the fluorescent markers DiI/DiO injected at ∼20-50 sites in limb mesenchyme. To calculate the D-V growth rate, the thickness of the limb buds was measured with an OPT scanner 3001 (see supplementary material Appendix S2 for details).

### Measurement of cell-cycle time

Cell-cycle time was measured by pulse-chase labeling with BrdU/IddU (Martynoga et al., 2005) (see supplementary material Appendix S5 for details).

### Measurement of SHH signaling activity using a luminescent system

We visualized and quantified Gli-binding site-fused luciferase activity derived from SHH signaling, using an Olympus Luminoview 200 and the Chroma-Glo Luciferase Assay System (Promega) (see supplementary material Appendix S6).

### Bead implantation experiments

AG1X-2 beads (Bio-Rad) soaked with 1 mg/ml SU5402, 1 mg/ml cyclopamine or 2 mg/ml SP600125 were used (Suzuki et al., 2004). SP600125 (Wako) was also added directly over the hindlimb with 45% HBC (Sigma) (Nissim et al., 2007). We examined the effects of beads in the posterior and distal zones by quantifying the average change in tissue growth rate and deformation anisotropy in the region within 200 µm from the center of the bead implanted in each zone (see also supplementary material Fig. S3).

### Inhibition of cell proliferation with trichostatin

Several very small DiI crystals were implanted in the distal region of the hindlimb at stage 22. Directly over the limb, 20 µl of 50 µg/ml trichostatin A or DMSO/PBS solution were added. Following incubation for 24 h, embryos were harvested and the deformation anisotropy was calculated from the relative positional changes of four DiI markers (supplementary material Appendix S8).

## Supplementary Material

Supplementary Material
