# Supplementary material for: Quantitative analysis of tissue deformation dynamics reveals three characteristic growth modes and globally aligned anisotropic tissue deformation during chick limb development
Source: Development. 2015 May 1;142(9):1672–83. doi: 10.1242/dev.109728 (PMC4419272; doi:10.1242/dev.109728)
Supplement: Supplementary Material [file supp_142_9_1672__index.html]

Supplementary Material 

# Quantitative analysis of tissue deformation dynamics reveals three characteristic growth modes and globally aligned anisotropic tissue deformation during chick limb development

## DEV109728 Supplementary Material

**Files in this Data Supplement:**

- Supplementary Material
