# Supplementary material for: Quantitative analysis of tissue deformation dynamics reveals three characteristic growth modes and globally aligned anisotropic tissue deformation during chick limb development
Source: Development. 2015 May 1;142(9):1672–83. doi: 10.1242/dev.109728 (PMC4419272; doi:10.1242/dev.109728)
Supplement: Supplementary Material [file supp_dev.109728_DEV109728supp.pdf]

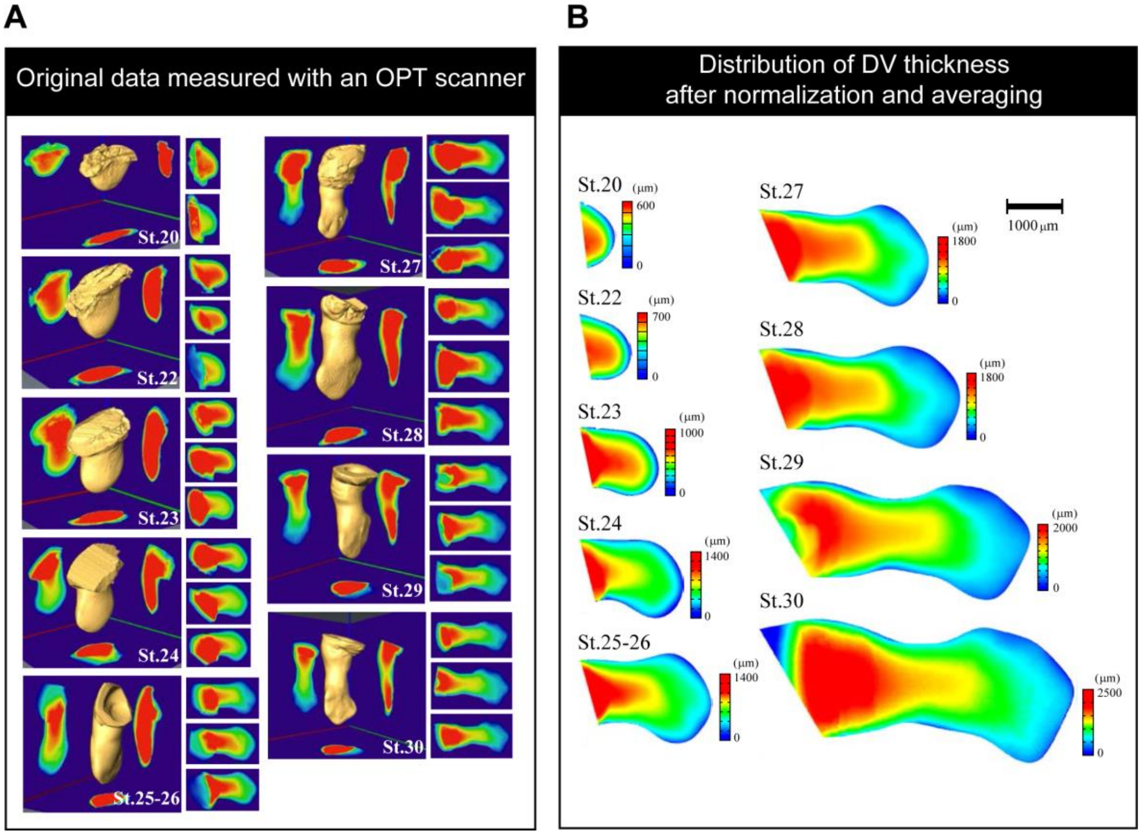

Figure S1: (A) Raw data showing spatial distribution of dorso-ventral limb bud thickness measured with an OPT scanner across 9 consecutive developmental stages (St.20-St.30). (B) Average distribution of DV thickness.

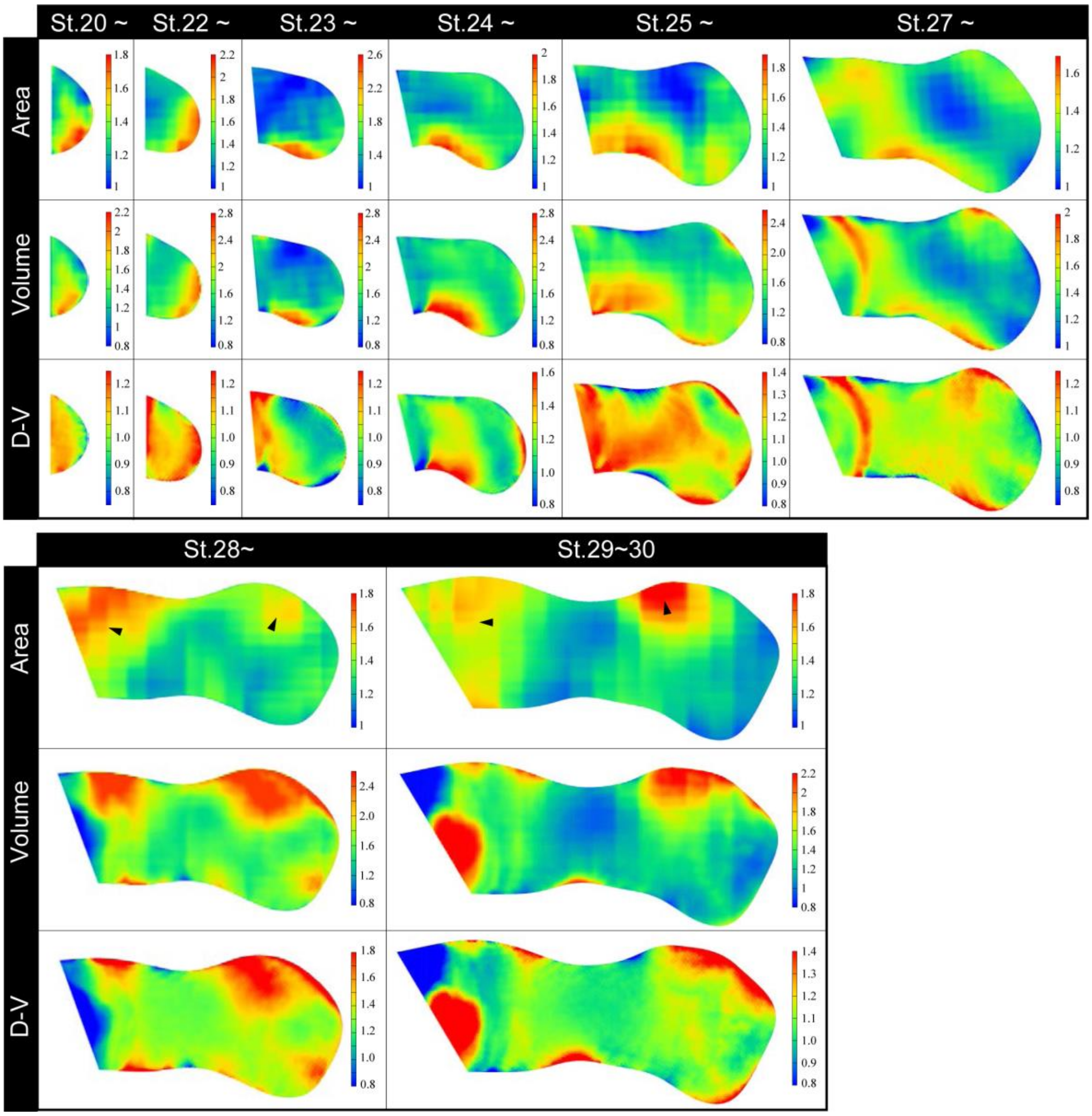

Figure S2: Spatio-temporal patterns of area (top), volume (middle), and DV growth rates (bottom) from St.20-St.30. The area and volume growth patterns are very similar to each other, and thus we use the term “tissue growth rate” to refer to both of them in the main text. In contrast, the correlation between the area and DV growth rate is low (-0.1~0.4) (Figure 3B), demonstrating that 2D deformation in the frontal plane and DV growth can be dealt with independently, at least during chick hindlimb development.

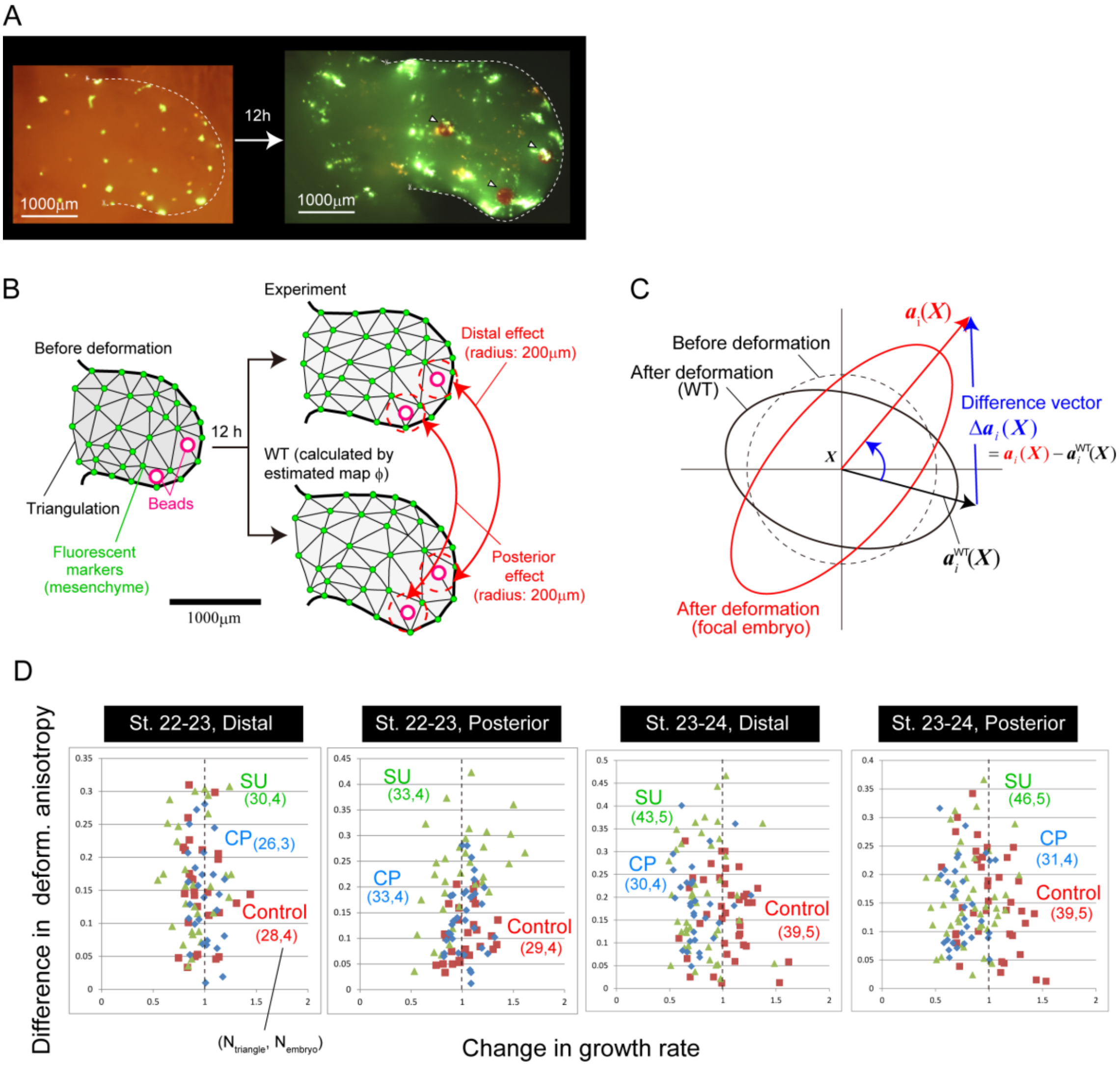

Figure S3: Implantation experiments using beads soaked with inhibitors of FGF (SU5402) and SHH (cyclopamine). Most beads were implanted in the posterior and/or distal sides of the right limb bud. In some embryos, beads were also implanted in the middle of the right limb bud. The left limb bud was used for the normalization of limb bud size to remove embryo-to-embryo variability. We performed the implantation experiment for two consecutive time intervals (St. 22-23 and St. 23-24). (A) As in the estimation of the deformation map, a few tens of DiI and DiO fluorescent markers were injected into the mesenchyme of each limb bud. The arrow heads indicate implanted beads. (B) Each limb bud was divided into multiple triangles based on the marker positions. From the relative positional changes of the markers, we calculated the area growth rate and deformation anisotropy vector for each triangle in each limb bud. We then compared the spatial patterns of growth rate and deformation anisotropy with those

of the wild type limb buds, which were calculated by using the distribution of fluorescent markers before deformation and the 2D estimated map ϕ^{2D}. (C) To quantify the effects of inhibiting signaling on deformation anisotropy, "difference in deformation anisotropy" defined as $\left\| \boldsymbol{a}_i(\boldsymbol{X}) - \boldsymbol{a}_i^{\mathrm{WT}}(\boldsymbol{X}) \right\|$ was used, where $\boldsymbol{a}_i(\boldsymbol{X}) \equiv (1 - \lambda_{2,i} / \lambda_{1,i}) \boldsymbol{\nu}_{1,i} / \left\| \boldsymbol{\nu}_{1,i} \right\|$ and $\boldsymbol{a}_i^{WT}(\boldsymbol{X}) \equiv (1 - \lambda_{2,i}^{WT} / \lambda_{1,i}^{WT}) \boldsymbol{\nu}_{1,i}^{WT} / \left\| \boldsymbol{\nu}_{1,i}^{WT} \right\|$ represents anisotropy vectors at $\boldsymbol{X}$ for the i-th bud and for the wild type. λ and $\boldsymbol{\nu}$ are eigenvalues and eigenvectors of deformation gradient tensor (see Materials and Methods). The reason we use this index for quantifying the effect on the anisotropy is that this value tends to be larger in the case where the differences in the orientations of both vectors and the magnitudes of the vectors are larger. In the region where the anisotropy is small, its orientation is sensitive to various randomness, such as embryo-to-embryo variability of tissue deformation dynamics and measurement noise, which should not be included in the evaluation of the effects of inhibiting SHH or FGF signal on deformation anisotropy. (D) Distributions of "change in tissue growth rate" and "difference in deformation anisotropy" calculated for different triangles for different zones (distal or posterior) and different time intervals (St. 22-23 or St. 23-24). The graphs in Fig. 6B were made based on these distributions. The change in tissue growth rate is defined as the ratio of the growth rate with beads to that in the control case.

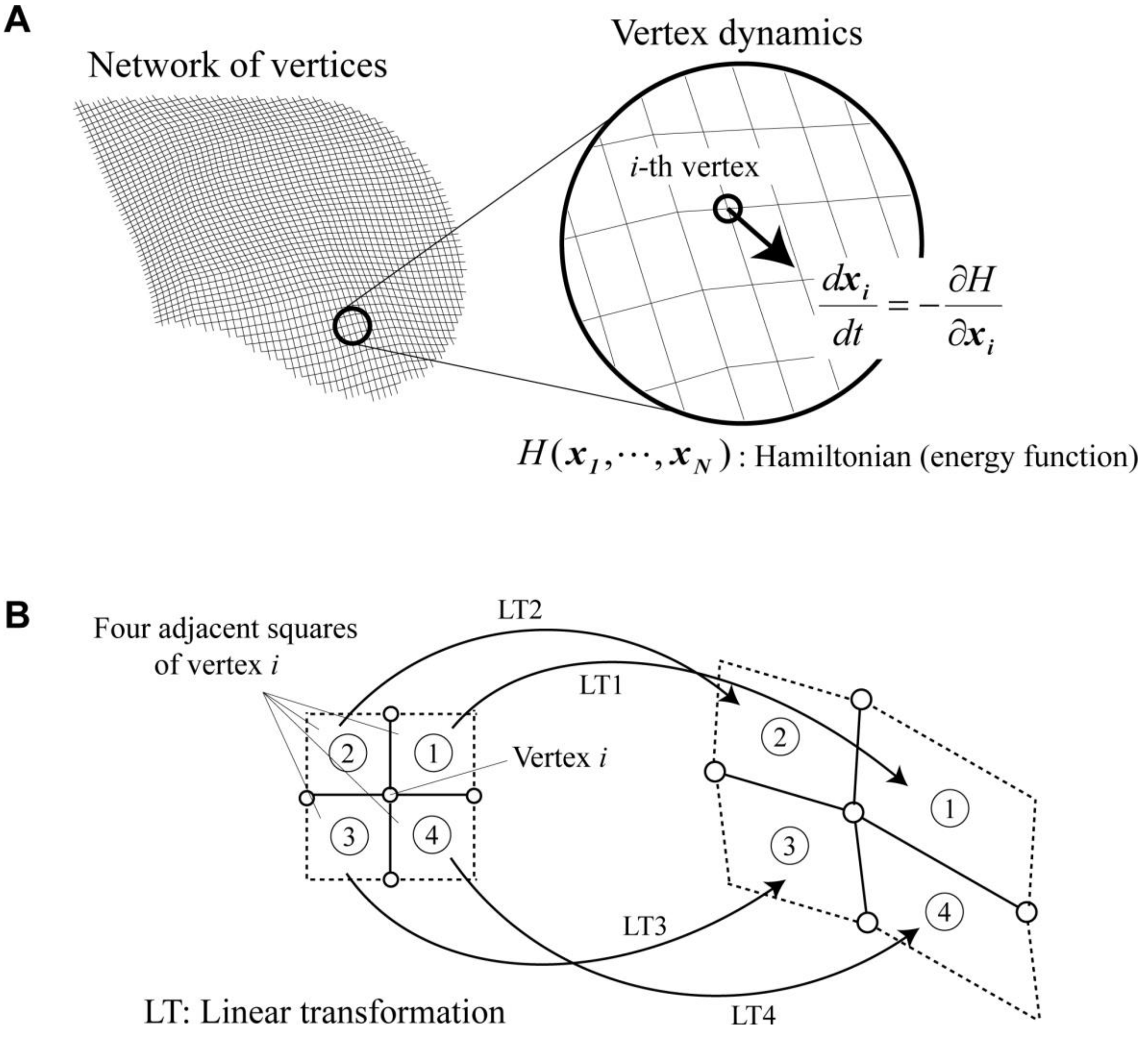

Figure S4: Scheme of a modified vertex dynamics model
(A) In our model, limb bud morphology is represented by a network of vertices; specifically, each piece of tissue is represented by a tetragon formed by linking four vertices. Each vertex moves so as to decrease the generalized energy of the system, called the Hamiltonian (see Supplementary Materials Appendix S7). (B) The Hamiltonian includes the deformation gradient tensor at each vertex i. Its matrix representation $F(i)$ is defined as the average of four linear transformations (LT1~LT4) for the deformations of four adjacent squares of the focal vertex i.

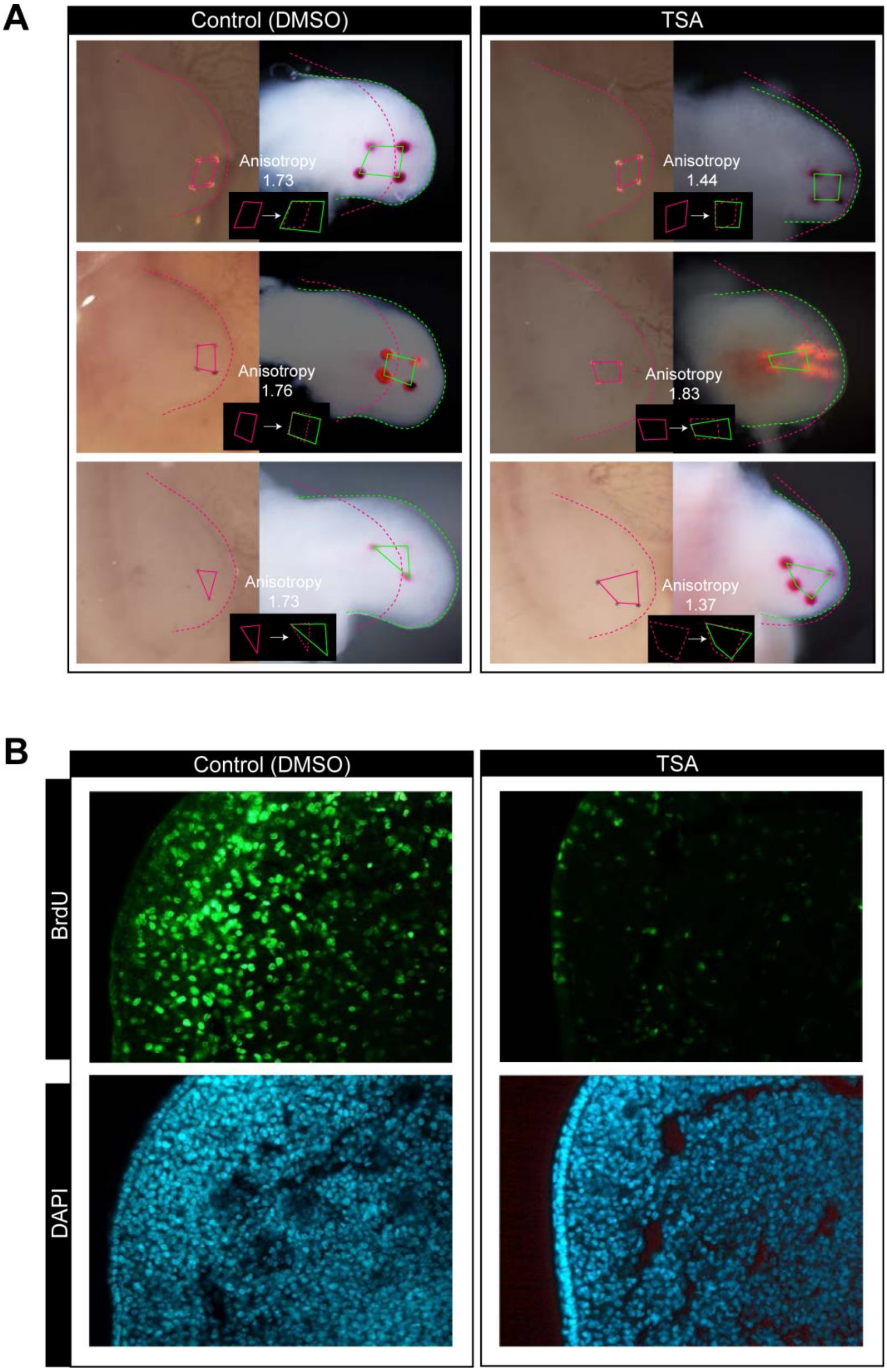

Figure S5: All data for the embryos treated with DMSO or trichostatin A.

(A) (left) embryos treated with DMSO (control) and (right) embryos treated with trichostatin A. (B) Inhibition of cell proliferation by Trichostatin A. Each embryo was

treated with 20 μl of 50 μg /ml Trichostatin A or DMSO, and cell proliferation was checked by BrdU incorporation after 24 hours. Immunohistochemistry was performed by using anti-BrdU staining. In the control embryos treated with DMSO, 41.0% of the posterior cells were in the S phase. In contrast, only 6% of the cells were in the S phase when embryos were treated with Trichostatin A ($p<0.005$).

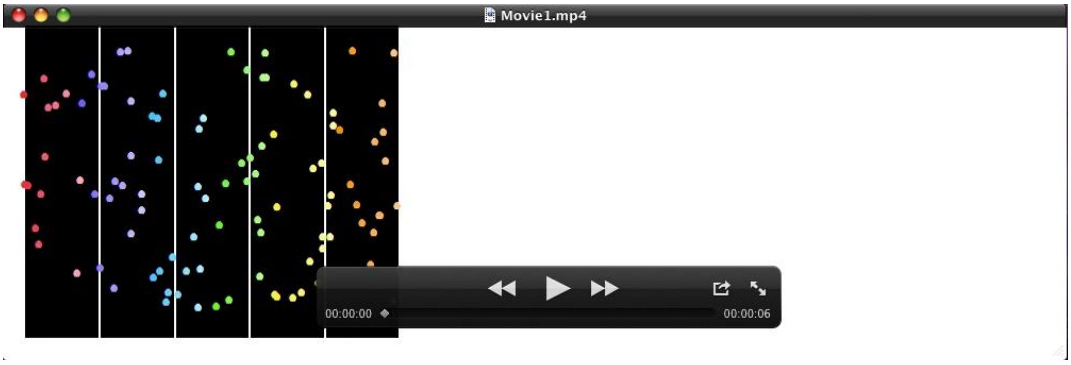

Movie 1: Randomly-labelled cells in a uniformly growing tissue with the camera fixed at the leftmost of tissue. In all movies, small noise was added to the position or trajectory of each labelled cell.

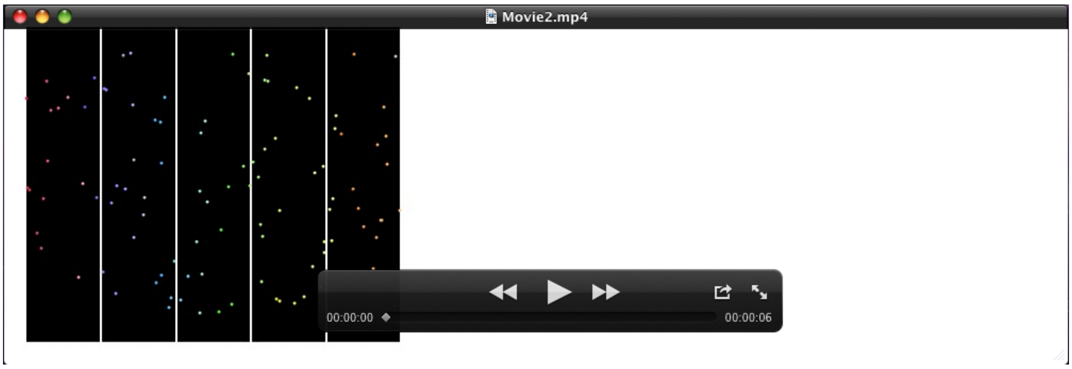

Movie 2: Cell trajectories in a uniformly growing tissue with the camera fixed at the leftmost of tissue.

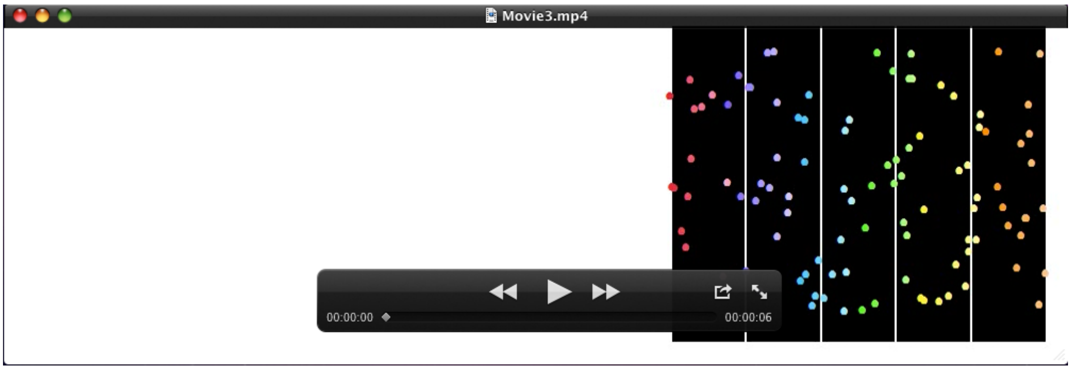

Movie 3: Randomly-labelled cells in a uniformly growing tissue with the camera fixed at the rightmost of tissue.

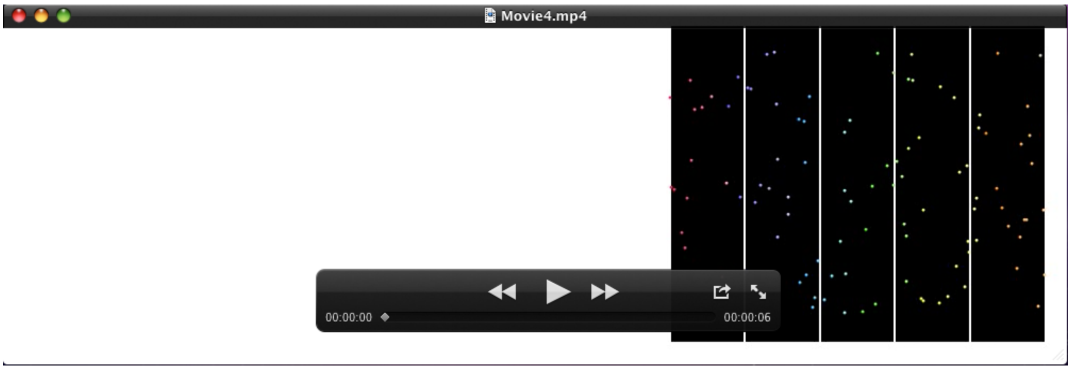

Movie 4: Cell trajectories in a uniformly growing tissue with the camera fixed at the rightmost of tissue.

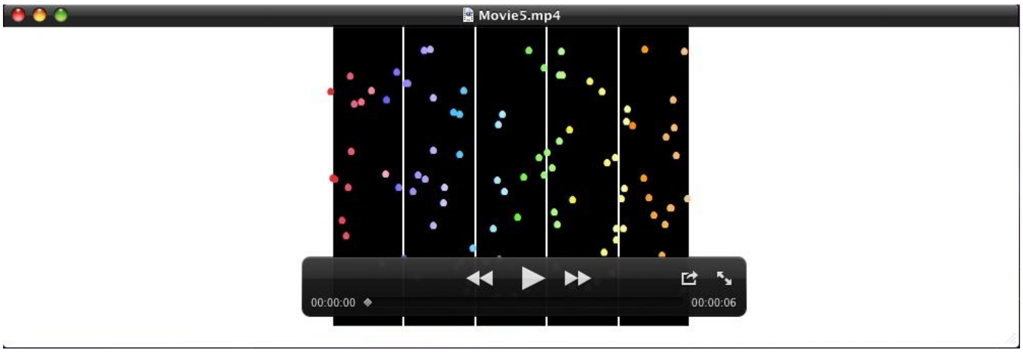

Movie 5: Randomly-labelled cells in a uniformly growing tissue with the camera fixed at the center of tissue.

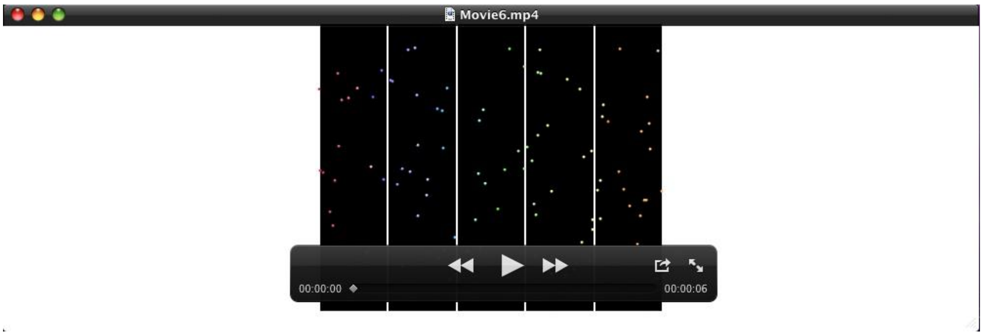

Movie 6: Cell trajectories in a uniformly growing tissue with the camera fixed at the center of tissue.

Appendices

S1. Mathematical details for Fig. 1F

The 1D tissue growth dynamics is given by the following equation:

$$x(X,t)=\int_0^X \exp\left[\int_0^t s(\xi,\tau)d\tau\right]d\xi ,$$

where $x(X,t)$ is the position at time t of the cell that is initially located at X. $s(X,t)$ is the tissue growth rate that the cell X experiences at time t. In this equation, the coordinate of the leftmost (or the proximal end) is set to be 0. In Fig. 1F, for simplicity of explanation, we assumed spatially and temporally uniform growth, i.e., $s(X,t)=s=\mathrm{constant}$, so that the growth dynamics is simply given by $x(X,t)=X\exp(st)$. Next, let us consider the camera position. When the camera is fixed at the proximal end, since the position of each cell X relative to that of the camera (denoted by $\tilde{x}_{\mathrm{LEFT}}(X,t)$) is equal to $X\exp(st)$, the velocity of the cell is observed as:

$$v_{\mathrm{LEFT}}(X,t)=\frac{\partial}{\partial t}\tilde{x}_{\mathrm{LEFT}}(X,t)=\frac{\partial}{\partial t}X\exp(st)=sX\exp(st).$$

Therefore, in this case, the velocity becomes higher for cells located farther to the right or more distally. It should be noted that this does not mean that the motility of cells located at the distal side is higher because uniform-growth is assumed. This example shows that tissue deformation results in apparent movement of cells. On the other hand, when the camera is fixed at the distal end (i.e., the rightmost), the relative position is given as:

$$\tilde{x}_{\mathrm{RIGHT}}(X,t)=X\exp(st)-L_0\exp(st),$$

where L_0 is the initial length of the limb bud. Thus the velocity is:

$$v_{\mathrm{RIGHT}}(X,t)=s(X-L_0)\exp(st).$$

In this case, the velocity becomes higher for cells located farther to the left or more proximally. Again, this does not mean that the motility of cells located at the proximal side is higher. It should be noted that the limb bud does not grow inside the body, although the velocity of cells on the proximal side looks faster. In the case where the camera is fixed so as to focus on the cell located at the center of the tissue, the following holds:

$$\tilde{x}_{\mathrm{CENTER}}(X,t)=X\exp(st)-\frac{L_0}{2}\exp(st),$$

$$v_{\mathrm{CENTER}}(X,t)=s(X-\frac{L_0}{2})\exp(st).$$

In this case, the velocity becomes maximal at the two ends of the tissue.

Finally, we note that the above argument holds not only for uniform growth, but also for arbitrary non-uniform growth dynamics.

S2. Data for quantifying tissue deformation dynamic (details)

(i) DiI and DiO markers on the frontal plane (spanned by the P-D and A-P axes).

We injected the fluorescent marker DiI/DiO at approximately 20-50 sites in limb mesenchyme at a depth of about 100 μm from the dorsal ectoderm using a sharp tungsten needle that had been dipped in a 1 mg/ml DiI/DiO solution (Fig. 2B). We used only limb buds of the same length at each stage by measuring with an ocular micrometer. Fluorescent images were taken using a Leica M205FA microscope with a digital zoom focus to adjust the precise magnification every time. The diameter of each marker was about 50 μm. To estimate the 2D deformation map for each time interval, we used marker positional data from 7 or 8 embryos (representing a few hundred markers in total). Since limb bud size was slightly different between embryos, data for each embryo were normalized along the PD and AP axes.

(ii) Measurement of dorso-ventral thickness with an OPT scanner

The thicknesses of the limb buds along the dorso-ventral axes were measured with an OPT (optical projection tomography) scanner 3001 (Sharpe et al., 2002). The shape of each limb bud was captured by detecting autofluorescence of the limb bud embedded in 1% low melting point agarose diluted in PBS. To avoid shrinkage and deformation of the tissue due to dehydration, samples were not cleared with Benzyl benzoate/Benzyl alcohol. Data on DV thickness were extracted by Avizo software.

S3. Validation of the estimated deformation map for chick hindlimb development

We evaluated the accuracy of the estimated deformation maps by their predictive performances (cross-validation) (Figs. 2F, G). For each time interval, 90% of the positional data for injected fluorescent markers before and after deformation was used for estimating the deformation maps by the proposed Bayesian method, and the remaining 10% of data was used for calculating the predictability that was evaluated by the mean square error of predicted positions of markers after deformation. The error was calculated along the proximo-distal and antero-posterior axes separately as follows:

$$\text{Error along P-D axis:} \quad \delta x = \sqrt{\left(\sum_{i=1}^{N_p} \left\| x_i^{(\text{Data})} - \hat{x}_i \right\|^2 \right) / N_p} \ ,$$

$$\text{Error along A-P axis:} \quad \delta y = \sqrt{\left(\sum_{i=1}^{N_p} \left\| y_i^{(\text{Data})} - \hat{y}_i \right\|^2 \right) / N_p} \ ,$$

where $(x_i^{(\text{Data})}, y_i^{(\text{Data})})$ is the positional data of the i-th marker. N_p is the number of

data points used for calculating the predictability.

S4. Correlation analysis between area and DV growth rates

In Fig. 3B, for the different time intervals, we calculated correlation coefficients between area and DV growth rates, ρ_{AD}, as follows:

$$\rho_{\mathrm{AD}} = \mathrm{E}[(A(\boldsymbol{x}_i) - E_A)(D(\boldsymbol{x}_i) - E_D)]/(\sigma_A \sigma_D),$$

where $A(\boldsymbol{x}_i)$ and $D(\boldsymbol{x}_i)$ are the area and DV growth rates at each lattice point $\boldsymbol{x}_i$, respectively (see also Fig. S2). E_A and E_D are their averages over the whole limb bud. σ_A and σ_D are their standard deviations. The correlation between area and DV growth is low ($\rho_{AD} = -0.1 \sim 0.4$) although there are a few stages in which the spatio-temporal patterns look similar.

S5. Measurement of cell cycle time (details)

Cell cycle time was measured by pulse-chase labeling with BrdU/IddU (Martynogaa et al., 2005). First, 100 μl of IddU (100 mg/ml) was injected into the yolk sac, and embryos were incubated at 38.6^{o}C for 1.5 h. Next, 100 μl of BrdU (100 mg/ml) was injected into the yolk sac, and embryos were incubated for another 30 min, followed by fixation with 4% paraformaldehyde. Immunohistochemistry of BrdU and IddU was previously described (Boehm et al., 2010). When the difference in the labeling time between IddU and BrdU is 1.5 h, cell cycle time can be calculated by the following equation:

(Cell cycle time) (h) = (Total number of cells) $\div$ (number of cells labeled only with BrdU) $\times$ 1.5

For three consecutive developmental stages (St.22, St.23, and St.24), we measured the cell cycle time at three regions: posterior, distal, and middle (Figure 4). For each region, we chose a spatial window of $100\mu m \times 100\mu m \times 7\mu m$ (DV thickness), and the cell cycle time for each region and stage was averaged over three different embryos.

S6. Measurement of SHH signaling activity using a luminescent system (details)

The Gli-responsive element (Sasaki, H., et al., *Development* 124, 1313-1322, 1997) was fused to the emerald luciferase gene (Toyobo) and inserted into the RCANBP(A) vector to detect SHH signaling activity by measuring emerald luciferase activity. To enable high temporal resolution, a PEST sequence was included at the C-terminal end to destabilize the emerald luciferase protein. For normalization of emerald luciferase activity, we designed a construct with a red Phrixothrix luciferase gene (Promega) driven by the CMV promoter in the RCANBP(B) vector. After retroviral infection of the future hindlimb field of St. 11 chick embryos, we harvested each limb bud in Tyrode solution. The hindlimb bud was excised using a sharp tungsten needle, and the limb bud was placed in a 35-mm glass bottom dish filled with DMEM, 10%FBS, 2% chick serum,

and 1mM luciferin. The dish was incubated at 37°C in 5% CO_2. Individual luminescent wavelengths were visualized and quantified using an Olympus Luminoview 200 and the Chroma-Glo™ Luciferase Assay System (Promega) following the manufacturer's protocol. After measuring luminescence activity, we converted the values into numbers of photons per second using the unique quantum efficiency of the EM-CCD camera. After normalization of emerald luciferase activity to red Phrixothrix luciferase activity, we visualized and quantified SHH signaling activity in the limb bud using MetaMorph software.

S7. *In silico* experiment (a modified vertex dynamics model)

To determine if spatially-biased growth or deformation anisotropy is the greater contributor to limb-specific morphology and limb elongation, we performed *in silico* experiments for two different situations.

(I) The situation in which the spatial pattern of tissue growth rate is the same as that in normal development but the anisotropy is low over the whole limb bud.

(II) The situation in which the spatial pattern of anisotropy is similar to that in normal development but the tissue growth rate is uniform.

In order to obtain more significant differences in morphology, the simulation time interval was set at 24 hours: (A) St.20-St.23, (B) St.23-St.25, (C) St.25- St.28, (D) St.28-St.30 (Fig. 8). To simulate morphologies for both situations (I) and (II), we developed a modified vertex dynamics model (Supplementary Fig. S4A). In our model, each piece of tissue is represented by a tetragon formed by linking four vertices. Each vertex moves so as to decrease energy of the whole system, called the Hamiltonian (denoted by H):

$$\frac{d\boldsymbol{x}_i}{dt} = -\frac{\partial H(\boldsymbol{x}_1, \boldsymbol{x}_2, \cdots, \boldsymbol{x}_N)}{\partial \boldsymbol{x}_i},$$

where $\boldsymbol{x}_i$ is the spatial coordinate of vertex i ($i = 1, \cdots, N$) and t is the time. Depending on the Hamiltonian adopted, the dynamics of vertices change, leading to a different final morphology of the vertices network.

In our simulation, we used the Hamiltonian given by:

$$H = \sum_i \left(c_1 \left\| \det F(i) - \det F^{\text{target}}(i) \right\|^2 + c_2 \sum_{j,k} \left\| F_{jk}(i) - F_{jk}^{\text{target}}(i) \right\|^2 \right),$$

where the first and second term on the right hand side are the constraints of volume growth rate and tensor components at vertex i, respectively. $F(i)$ and $F_{jk}(i)$ are the matrix representations of the deformation gradient tensor **F** at i (for a given coordinate system) and its (j,k)-component, respectively. $F(i)$ is defined as the average of four linear transformations for the deformations of four adjacent squares (Supplementary Fig. S4B). $\det F^{\text{target}}(i)$ is the target volume growth rate. In simulation (I), it is set to be the same value as the wild type at each location (i.e., $\det F^{\text{target}}(i) = \det F^{WT}(i)$), and in simulation (II) it is set to be a constant, reflecting the average growth rate over the whole limb bud in the wild type (i.e.,

$\det F^{\text{target}}(i) = \mathrm{E}[\det F^{\text{WT}}]$). $F_{jk}^{\text{target}}(i)$ is the target tensor component. We adopted $F_{jk}^{\text{target}}(i) = \sqrt{\det F^{\text{WT}}(i)} R_{jk}$ (R_{jk} : rotation tensor) in simulation (I). In simulation (II), it is set to be a rescaled value of the wild type's as follows:

$$F_{jk}^{\text{target}}(i) = \frac{\sqrt{\mathrm{E}[\det F^{\text{WT}}]}}{\sqrt{\det F^{\text{WT}}(i)}} F_{jk}^{\text{WT}}(i) .$$

c_1 and c_2 are the coefficients of constraints. Depending on the combination of those values, the balance of constraints in the Hamiltonian varies. We performed many simulations for different combinations and searched the value that satisfies conditions (I) and (II).

S8. Calculation of deformation anisotropy in the experiment of inhibiting cell proliferation with Trichostatin

We first calculated the deformation gradient tensor $\boldsymbol{F}$ from the relative positional changes of four DiI markers; concretely, the tensor was obtained by minimizing the mean square error,

$$\frac{1}{4}\sum_{i=1}^{4}\left((x_i - (F_{11}X_i + F_{12}Y_i))^2 + (y_i - (F_{21}X_i + F_{22}Y_i))^2\right),$$

where (X_i, Y_i) and (x_i, y_i) are the position vectors for each marker i before and after deformation, respectively, when the origins of coordinates were set to the centroids of tetragons before and after deformation, respectively. From the resulting $\boldsymbol{F}$, the deformation anisotropy was calculated as the ratio of eigenvalues of the stretch tensor $\boldsymbol{U}$.
